# Editorial: Nutrition and urological disorders: the crossroads of contemporary research and clinical perspective

**DOI:** 10.3389/fnut.2024.1394600

**Published:** 2024-04-24

**Authors:** Milica Zeković, Irena Krga, Uroš Bumbaširević

**Affiliations:** ^1^Centre of Research Excellence in Nutrition and Metabolism, Institute for Medical Research, National Institute of Republic of Serbia, University of Belgrade, Belgrade, Serbia; ^2^Nutrition Department, University of California, Davis, Davis, CA, United States; ^3^Clinic of Urology, University Clinical Center of Serbia, Belgrade, Serbia; ^4^Faculty of Medicine, University of Belgrade, Belgrade, Serbia

**Keywords:** nutrition, urology, kidney stone disease (KSD), urolithiasis, Prognostic Nutritional Index (PNI), Contrast-Induced Nephropathy (CIN), diet, obesity

Featuring substantial diversity concerning epidemiological patterns, etiological determinants, pathophysiological mechanisms, clinical presentation, preventative strategies, treatment modalities, and eventually therapeutic outcomes, urological disorders delineate a complex spectrum of both benign and malignant pathologies affecting the male and female urinary tract alongside the male reproductive system. Within the landscape of modern medical practice, this heterogeneous array of diseases imposes a considerable clinical, humanistic, and economic burden exacting a significant toll on patients, healthcare providers, and society at large, thus accentuating the necessity for meticulously conducted and innovative research initiatives aimed at enhancing and expanding the scientific comprehension within this multifaceted domain. The remarkable prevalence of urological clinical entities, accompanied by the intricate determinants shaping their impact and severity, mandates a collaborative, transdisciplinary scientific perspective, entailing the coordinated accumulation and integration of evidence and knowledge across disciplines. The reciprocal exchange and mutual reinforcement between urology and interconnected biomedical fields create a solid platform for the evolution from a predominantly disease-centric medical framework to a comprehensive, holistic and personalized healthcare model.

Considering the profound implications of nutrition in molding the trajectory of health and disease over the life course, the domain of nutrition research, rooted in the tenets of biomedical and behavioral sciences, assumes paramount importance in deciphering the complex interrelationships between dietary factors and the genesis, progression, and mitigation of varied pathologic conditions, encompassing those afflicting the genitourinary system. Moreover, the ongoing shift toward integrated healthcare models that blur traditional boundaries between historically distinct nutritional and pharmaceutical approaches calls for a robust and systematic effort to accumulate, evaluate, consolidate, and disseminate evidence reflecting these evolving paradigms.

At the juncture of urology and nutrition lies a myriad of pertinent topics, such as the role of nutrition in preventing and managing urolithiasis, alimentative chemoprevention in urological cancers, the tactical deployment of dietary interventions in uro-oncology survivorship and palliative care, and the cutting-edge utilization of nutraceuticals in urological treatments. This interdisciplinary nexus also investigates the delicate interplay between nutritional status in general and clinical outcomes in urological patients, whilst the uro-andrological facet scrutinizes the impact of nutrients on molecular aspects related to sperm quality and the role of diet as a modifiable determinant influencing male reproductive potential.

Three studies presented in this Research Topic tackled the complexities of nephrolithiasis, contributing complementary insights into the multifaceted interplay between diet, obesity, and micronutrients in kidney stone disease (KSD) development and risk. Initiating the discourse, Yang et al. harnessed a robust two-sample Mendelian randomization analytical approach, deploying 36 single nucleotide polymorphisms (SNPs) associated to eight distinct micronutrients as instrumental variables. Their study exposed a causal association between raised genetically determined concentrations of vitamin B12 and zinc with amplified KSD risk, adeptly circumventing the issues tied to confounders, reverse causality, and recall bias often encountered in conventional epidemiologic analyses. Residual limitations, mostly attributable to unaddressed animal protein pleiotropy and exclusive European demographic representation, signal the opportunity for future investigations armed with expanded datasets. Parallelly, Gui et al. and Yin et al. independently explored the National Health and Nutrition Examination Survey (NHANES) dataset spanning 2007–2018, casting light on divergent angles of the intricate relationship between obesity, diet, and kidney stones. Gui et al. explored obesity indicators through the lens of the weight-adjusted waist circumference index (WWI), exposing a consistent positive association with kidney stones across varying degrees of adjustment for confounders The non-linear positive correlation between WWI and nephrolithiasis became more evident as WWI rose, peaking at a threshold value of 11.02, signifying a pressing public health concern demanding attention. Hinting at differential vulnerabilities necessitating tailored interventions, subgroup analyses divulged interesting insights while acknowledging cross-sectional design constraints. Finally, the study authored by Yin et al. analyzed dietary recall data on added sugars and, controlling for various confounding variables, multivariable and stratified logistic regression revealed strong positive correlation between greater percentages of energy intake from added sugars and heightened kidney stone incidence. Offering valuable cross-sectional evidence, this study sets the stage for further research or more high-quality prospective studies to affirm causality and patch the gaps left by the study's constraints. Altogether, this triptych of research propelled a more comprehensive understanding of the manifold etiologies behind nephrolithiasis, guiding scientists and clinicians toward a more targeted, inclusive approach in risk management.

Going further with featured articles in this Research Topic, Chang et al. conducted a comprehensive meta-analysis seeking to understand the relationship between the Prognostic Nutritional Index (PNI) and the risk of Contrast-Induced Nephropathy (CIN) in patients exposed to coronary angiography. It unveiled a higher risk of CIN in patients presenting with low PNI scores, and the increased occurrence of CIN subsequently translated to amplified mortality risk. Showcasing major ramifications for optimizing CIN risk factor detection, presented findings placed malnutrition as a crucial factor in the emergence of this severe complication. Accordingly, early nutritional assessment in patients exposed to nephrotoxic agents, such as contrast medium, becomes paramount, particularly in high-risk groups. Strengthening of these revelations calls for well-executed randomized controlled trials that aim to deepen our comprehension of malnutrition's influence on CIN risk, ultimately contributing to the refinement of therapeutic decision-making processes.

As a valuable resource hub for scientists, scholars, and medical practitioners, this Research Topic promotes the systematic appraisal, discussion, and navigation of present knowledge, charting courses for future perspectives while addressing emerging challenges, controversies, and opportunities at the confluence of nutrition and urology. The assembled body of research illustrates the immense potential for synergistic collaboration and cross-pollination of concepts, techniques, and experiences, thereby accelerating progress in understanding and combating urological disorders. Such convergence of expertise may not only create unique opportunities and novel avenues of investigation, but also serve as a catalyst for innovation, propelling data-driven advancements and informing patient-care trends within urology.

## Author contributions

MZ: Conceptualization, Writing – original draft, Writing – review & editing, Project administration. IK: Conceptualization, Writing – original draft, Writing – review & editing, Project administration. UB: Conceptualization, Writing – original draft, Writing – review & editing, Project administration.

